# ROS scavenging Mn_3_O_4_ nanozymes for *in vivo* anti-inflammation†
†Electronic supplementary information (ESI) available: Additional figures and associated discussions. See DOI: 10.1039/c7sc05476a


**DOI:** 10.1039/c7sc05476a

**Published:** 2018-02-16

**Authors:** Jia Yao, Yuan Cheng, Min Zhou, Sheng Zhao, Shichao Lin, Xiaoyu Wang, Jiangjiexing Wu, Sirong Li, Hui Wei

**Affiliations:** a Department of Biomedical Engineering , College of Engineering and Applied Sciences , Nanjing National Laboratory of Microstructures , Nanjing University , Nanjing , Jiangsu 210093 , China . Email: weihui@nju.edu.cn ; http://weilab.nju.edu.cn ; Fax: +86-25-83594648 ; Tel: +86-25-83593272; b State Key Laboratory of Analytical Chemistry for Life Science , School of Chemistry and Chemical Engineering , Collaborative Innovation Center of Chemistry for Life Sciences , Nanjing University , Nanjing , Jiangsu 210023 , China

## Abstract

Reactive oxygen species (ROS)-induced oxidative stress is linked to various diseases, including cardiovascular disease and cancer. Though highly efficient natural ROS scavenging enzymes have been evolved, they are sensitive to environmental conditions and hard to mass-produce. Therefore, enormous efforts have been devoted to developing artificial enzymes with ROS scavenging activities. Among them, ROS scavenging nanozymes have recently attracted great interest owing to their enhanced stability, multi-functionality, and tunable activity. It has been implicated that Mn-contained nanozymes would possess efficient ROS scavenging activities, however only a few such nanozymes have been reported. To fill this gap, herein we demonstrated that Mn_3_O_4_ nanoparticles (NPs) possessed multiple enzyme mimicking activities (*i.e.*, superoxide dismutase and catalase mimicking activities as well as hydroxyl radical scavenging activity). The Mn_3_O_4_ nanozymes therefore significantly scavenged superoxide radical as well as hydrogen peroxide and hydroxyl radical. Moreover, they were not only more stable than the corresponding natural enzymes but also superior to CeO_2_ nanozymes in terms of ROS elimination. We showed that the Mn_3_O_4_ NPs not only exhibited excellent ROS removal efficacy *in vitro* but also effectively protected live mice from ROS-induced ear-inflammation *in vivo*. These results indicated that Mn_3_O_4_ nanozymes are promising therapeutic nanomedicine for treating ROS-related diseases.

## Abstract 2

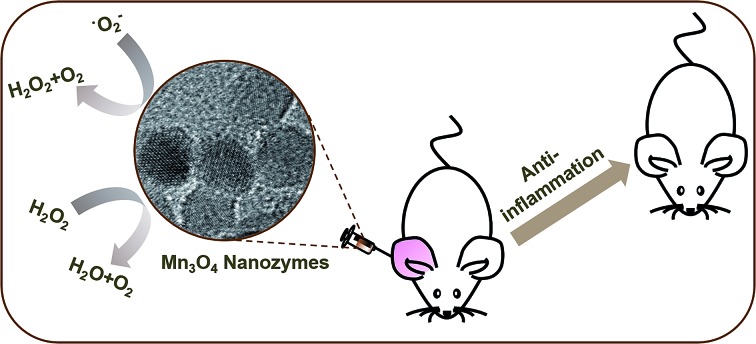
Reactive oxygen species (ROS) scavenging Mn_3_O_4_ nanozymes effectively protected live mice from ROS-induced ear-inflammation *in vivo*.

## Introduction

Inflammation has been demonstrated to cause various diseases, such as rheumatoid arthritis,^1^ cardiovascular diseases,^2^ and even cancer.^3^ It has been established that the dysregulation of highly active reactive oxygen species (ROS), including superoxide radical (˙O_2_^–^), hydrogen peroxide (H_2_O_2_) and hydroxyl radical (˙OH), plays very important roles in these diseases.^4^ The dysregulated ROS can lead to oxidative stress, causing harm to biomolecules like DNA, proteins and lipids.^5^,^6^ Therefore, live organisms have evolved a number of antioxidant enzymes to scavenge ROS and protect tissues from inflammation-induced damages.^7^,^8^ Among them, superoxide dismutase (SOD) catalyzes the dismutation of ˙O_2_^–^ to H_2_O_2_. Subsequently, catalase catalyzes the reduction of H_2_O_2_ to water.^9^ Though ROS scavenging natural enzymes work efficiently to combat inflammation, they are sensitive to environmental conditions (such as temperature and pH value) and hard to mass-produce.^10^ Therefore, intensive efforts have been made to develop ROS scavenging artificial enzymes to overcome the inherent drawbacks of the natural enzymes.^11^,^12^

In last decades, catalytic nanomaterials with natural enzyme-like activities (termed as nanozymes) have been developed as emerging artificial enzymes.^13^–^31^ Nanozymes have many advantages, such as excellent thermal and biological stability, multi-functionality, ease-of-preparation, and tunable activity.^13^,^32^–^34^ Among these developed nanozymes, a variety of nanomaterials with ROS scavenging activities have been reported.^35^–^38^ For instance, ceria nanoparticles (CeO_2_ NPs) have been demonstrated to possess SOD mimicking activities due to the mixed valance states of Ce^3+^ and Ce^4+^.^35^ Nevertheless, the ROS scavenging capability of most nanozymes is moderate. Therefore, numerous strategies have been proposed to design more active nanozymes. One possible strategy is adding dopant ions (such as Zr^4+^) into ceria NPs to modulate the ratio of Ce^3+^/Ce^4+^.^39^ The superoxide scavenging activity of ceria NPs could also be enhanced through an electron transfer strategy.^40^ On the other hand, pharmacokinetic studies revealed that natural Mn SOD is superior to Cu/Zn SOD and Fe SOD for chronic diseases treatment,^41^ which inspires a long effort to synthesize Mn based SOD mimics.^42^ Despite of the great promise, only a few Mn-based nanozymes with antioxidant activities have been developed so far.^43^–^45^ A very recent study reported the antioxidant activities of flower-like manganese oxide NPs and applied them for cellular protection *in vitro*.^45^ However, Mn-based nanozymes have not been explored for *in vivo* anti-inflammation yet.

To fill this gap, here we demonstrated that Mn_3_O_4_ NPs synthesized *via* a hydrothermal method possessed remarkable SOD mimicking activities, thanks to the double oxidation states of Mn^2+^ and Mn^3+^. Thus, the Mn_3_O_4_ NPs could be used to eliminate ˙O_2_^–^ by the disproportionation of ˙O_2_^–^ into H_2_O_2_ and O_2_. Besides, the Mn_3_O_4_ NPs were also capable to catalyze the elimination of H_2_O_2_ and scavenge ˙OH. Notably, the ROS scavenging capacity of Mn_3_O_4_ NPs was superior to CeO_2_ NPs, which promoted us to further apply them to eliminate ROS both *in vitro* and *in vivo*. We showed that the Mn_3_O_4_ NPs not only exhibited excellent ROS removal efficacy *in vitro* but also effectively protected live mice from ROS-induced ear-inflammation *in vivo*.

## Results and discussion

### Synthesis and characterization of Mn_3_O_4_ NPs

**Fig. 1:**
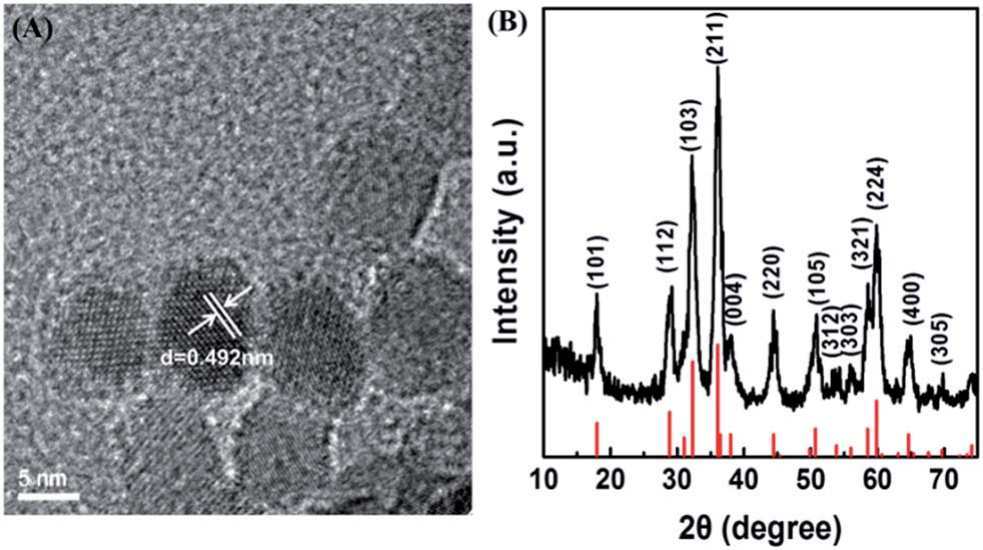
(A) TEM image of Mn_3_O_4_ NPs. (B) Powder X-ray diffraction pattern of Mn_3_O_4_ NPs (the red lines at the bottom mark the reference pattern of hausmannite Mn_3_O_4_ from the JCPDS database, card no. 24-0734).

The Mn_3_O_4_ NPs were synthesized by a hydrothermal method.^46^ TEM and XRD techniques were employed to analyze the morphology and the phase composition of Mn_3_O_4_ NPs. As presented in Fig. 1A and S1A,† the Mn_3_O_4_ NPs were uniform polyhedrons with an average size of 7–8 nm. Dynamic lighting scattering (DLS) (Fig. S2†) and zeta potential (Fig. S3†) results of the newly-prepared and two-month stored Mn_3_O_4_ NPs demonstrated their good long-term storage stability. The lattice shown in TEM agreed well with *d*_[101]_ = 0.492 nm, demonstrating highly crystalline nature of the as-prepared Mn_3_O_4_ NPs. The XRD pattern of Mn_3_O_4_ NPs was shown in Fig. 1B. All the measured diffraction peaks matched well to the standard pattern of hausmannite Mn_3_O_4_ [JCPDS card No. 24-0734, *I*4_1_/*amd* (141)], confirming their highly crystalline nature. As catalytic reactions happened on the surface of NPs, the surface of Mn_3_O_4_ NPs was further characterized by X-ray photoelectron spectroscopy (XPS) (Fig. S4†). Mn 2p_3/2_ and 2p_1/2_ peaks were centered at 641.7 eV and 653.5 eV, respectively. The area ratio (*i.e.*, molar ratio) of Mn^2+^ and Mn^3+^ was about 1 : 2, which agreed well with the theoretical value.

### SOD mimicking activity of Mn_3_O_4_ NPs

**Fig. 2:**
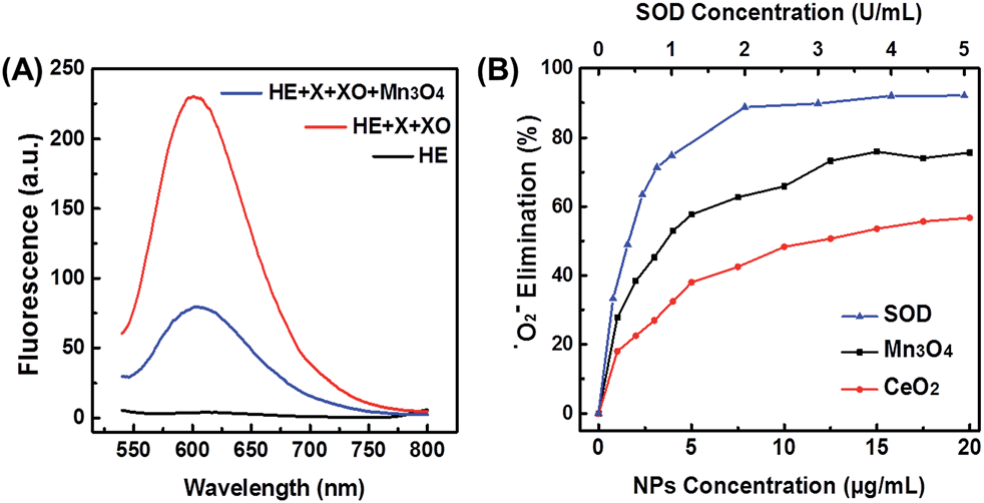
(A) Fluorescent spectra of HE after reaction with X and XO, in the absence and presence of Mn_3_O_4_ NPs. X for xanthine and XO for xanthine oxidase, respectively. (B) Dependence between the elimination of ˙O_2_^–^ and concentrations of Mn_3_O_4_ NPs, CeO_2_ NPs, and natural SOD.

Since ˙O_2_^–^ is one of the most destructive ROS, scavenging ˙O_2_^–^ by Mn_3_O_4_ NPs was first investigated. Such ˙O_2_^–^ scavenging activity could be attributed to the SOD mimicking activity of Mn_3_O_4_ NPs. First, ˙O_2_^–^ was generated by the reaction of xanthine and xanthine oxidase. Then the ability of Mn_3_O_4_ NPs to scavenge ˙O_2_^–^ was characterized by a ˙O_2_^–^ specific probe hydroethidine (HE). HE could react with ˙O_2_^–^ to produce fluorescent ethidium, which emitted strong fluorescence centered at 610 nm (Fig. 2A, red line). In the presence of Mn_3_O_4_ NPs, the fluorescent intensity was significantly reduced (Fig. 2A, blue line) compared with the red line, which illustrated the efficient elimination of ˙O_2_^–^ by Mn_3_O_4_ NPs. The SOD-like catalytic activity of Mn_3_O_4_ NPs was dose-dependent, and the ˙O_2_^–^ scavenging efficiency reached almost 75% when the concentration of Mn_3_O_4_ NPs was 20 μg mL^–1^. This result was comparable with that using 1 U mL^–1^ natural SOD (Fig. 2B), indicating the excellent SOD-like activity of Mn_3_O_4_ NPs. Besides, when compared with the most widely used SOD mimicking CeO_2_ nanozymes (Fig. S1B†), the Mn_3_O_4_ NPs exhibited 1.5-fold enhancement than CeO_2_ NPs under the same experimental condition (Fig. 2B). Moreover, Mn_3_O_4_ NPs showed better thermal stability than natural SOD (Fig. S5†). Mn_3_O_4_ NPs also possessed high SOD mimicking activities over a broad temperature range (from 20 to 60 °C), which has not been achieved for natural SOD (Fig. S6†). Two-month stored Mn_3_O_4_ NPs showed almost the same SOD mimicking activity as the newly-prepared NPs (Fig. S7†). In addition, the scavenging of ˙O_2_^–^ with Mn_3_O_4_ NPs was further confirmed by EPR with 5,5-dimethyl-1-pyridine N-oxide (DMPO) as a spin trap. As shown in Fig. S8,† the DMPO/˙OOH signal decreased with the increase of the concentration of Mn_3_O_4_ NPs, indicating the ability of Mn_3_O_4_ NPs to eliminate ˙O_2_^–^. These results illuminated that the Mn_3_O_4_ NPs showed significant SOD-like catalytic activity and high stability.

### Catalase mimicking activity of Mn_3_O_4_ NPs

**Fig. 3:**
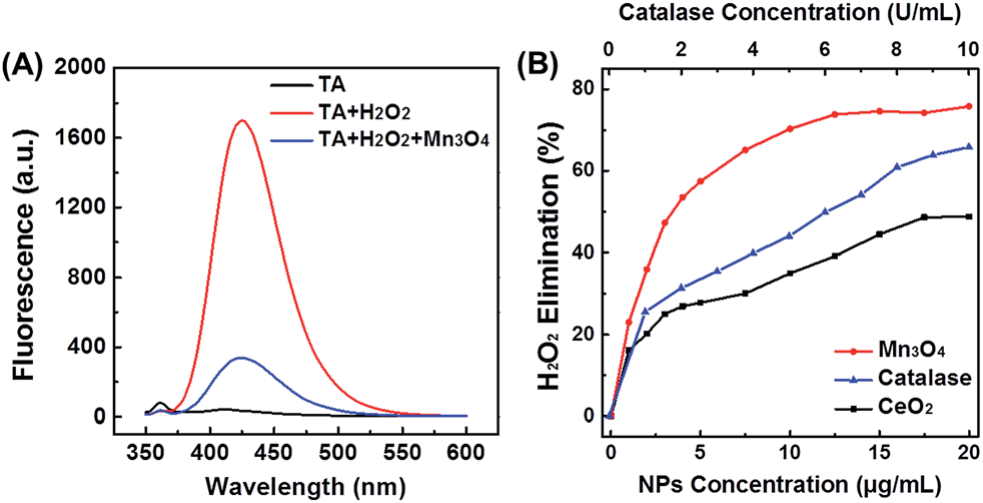
(A) Fluorescent spectra of TA after reaction with H_2_O_2_, in the absence and presence of Mn_3_O_4_ NPs. (B) Dependence between the elimination of H_2_O_2_ and concentrations of Mn_3_O_4_ NPs, CeO_2_ NPs, and natural catalase.

As H_2_O_2_ is the product of ˙O_2_^–^, another important ROS, the H_2_O_2_ elimination capacity of Mn_3_O_4_ NPs was also tested. As shown in Fig. 3A, terephthalic acid (TA) reacted with H_2_O_2_ to produce 2-hydroxyterephthalic acid with a fluorescent peak at 425 nm. In the presence of Mn_3_O_4_ NPs, the fluorescent intensity significantly decreased, illustrating the H_2_O_2_ elimination activity (*i.e.*, the catalase-like activity) of Mn_3_O_4_ NPs. The catalase-like of Mn_3_O_4_ NPs was concentration dependent, and about 75% of H_2_O_2_ was eliminated by using 20 μg mL^–1^ Mn_3_O_4_ NPs, which was even more efficient than that using 10 U mL^–1^ catalase. Besides, Mn_3_O_4_ NPs also showed better thermal stability than natural catalase (Fig. S10†). The H_2_O_2_ elimination capacity of Mn_3_O_4_ NPs was also higher than that of CeO_2_ NPs (Fig. 3B). In addition, the elimination of H_2_O_2_ with Mn_3_O_4_ NPs was also studied by monitoring the absorbance of H_2_O_2_ at 240 nm (Fig. S11†). Both the fluorescence and absorption spectra confirmed that Mn_3_O_4_ NPs could also eliminate H_2_O_2_ and possessed superior catalase mimicking property.

### Hydroxyl radical scavenging activity of Mn_3_O_4_ NPs

**Fig. 4:**
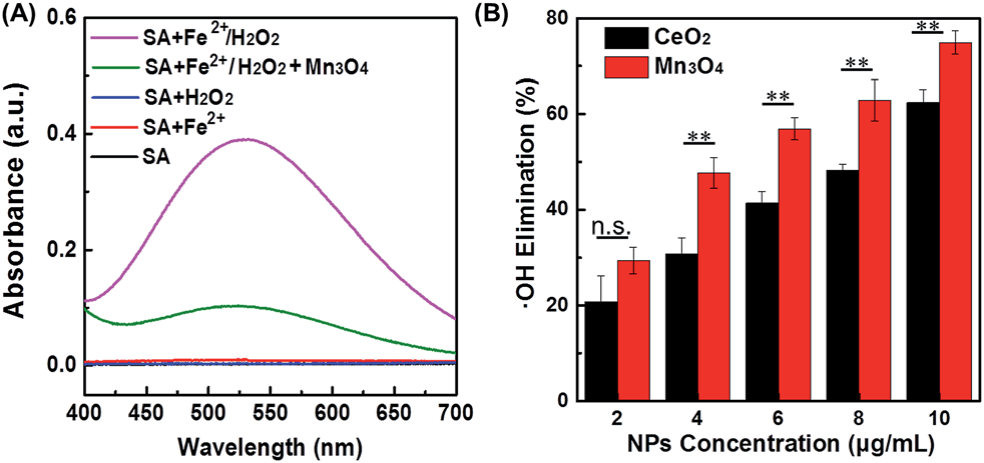
(A) Absorption spectra of SA after reaction with Fe^2+^/H_2_O_2_, in the absence and presence of Mn_3_O_4_ NPs. SA alone, and SA reacted with Fe^2+^ or H_2_O_2_ were used as control. (B) Dependence between the elimination of ˙OH and concentration of Mn_3_O_4_ NPs and CeO_2_ NPs (mean ±S.D., **p < 0.05; n.s., not significant).

˙OH is another important ROS, therefore its scavenging would help to protect cells or live organisms from ROS-induced damages more efficiently. To check whether the Mn_3_O_4_ NPs could also eliminate ˙OH, both absorption and EPR spectra were applied to detect ˙OH level in the presence of Mn_3_O_4_ NPs. Fenton reaction with the Fe^2+^/H_2_O_2_ system was used to produce ˙OH. First, the generated ˙OH was detected by its specific probe salicylic acid (SA). As shown in Fig. 4A, an obvious absorption peak at 510 nm was observed after mixing SA with Fe^2+^/H_2_O_2_. The absorption intensity decreased significantly after adding Mn_3_O_4_ NPs, which proved the ˙OH scavenging activity of Mn_3_O_4_ NPs. Fig. 4B showed that the elimination of ˙OH was enhanced with the increase of the NPs concentration. Nearly 70% elimination was achieved with 10 μg mL^–1^ of Mn_3_O_4_ NPs. We also compared Mn_3_O_4_ NPs with CeO_2_ NPs, demonstrating the higher ˙OH scavenging activity of Mn_3_O_4_ NPs (Fig. 4B). In addition, the ˙OH scavenging activity of Mn_3_O_4_ NPs was further confirmed by EPR. The signal of DMPO/˙OH decreased with the increase of Mn_3_O_4_ concentration, which was attributed to the scavenging of ˙OH (Fig. S12†). All the above results clearly demonstrated the highly efficient ROS scavenging activity of Mn_3_O_4_ NPs, which was better than the most widely used CeO_2_ nanozymes.

### Intracellular ROS scavenging detection

**Fig. 5:**
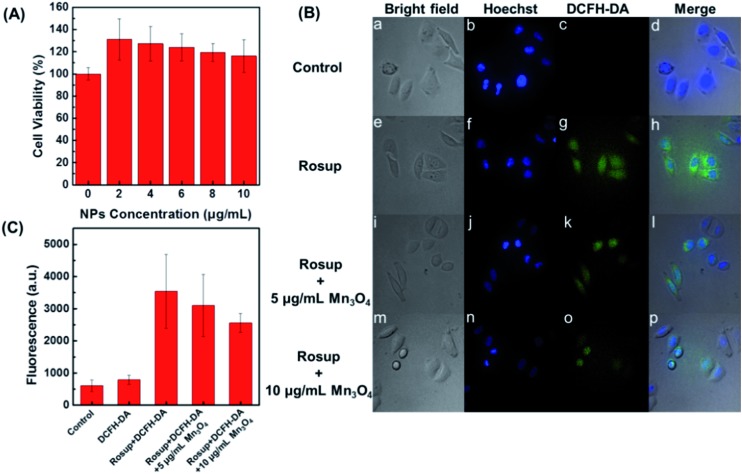
(A) Hela cell viability under different concentrations of Mn_3_O_4_ NPs. (B) Laser confocal fluorescence images of Hela cells with different treatments: (a–d) 0.01 mM DCFH-DA, (e–h) Rosup and DCFH-DA, (i–l) Rosup and DCFH-DA with 5 μg mL^–1^ Mn_3_O_4_ NPs, (m–p) Rosup and DCFH-DA with 10 μg mL^–1^ Mn_3_O_4_ NPs. (C) Corresponding fluorescent intensity of DCFH-DA in panel (B).

After demonstrating the scavenging abilities of Mn_3_O_4_ NPs toward ˙O_2_^–^, H_2_O_2_, and ˙OH, the intracellular ROS scavenging activity of Mn_3_O_4_ NPs was then investigated using Hela cell line as a model. First, the toxicity of Mn_3_O_4_ NPs was evaluated. The MTT results showed that the Mn_3_O_4_ NPs exhibited no obvious cytotoxicity within the experimental conditions (Fig. 5A). Then the intracellular ROS level was monitored using 2′,7′-dichlorofluorescein diacetate (DCFH-DA) as the fluorescent probe.^47^ As shown in Fig. 5B, the treatment with Rosup resulted in remarkable fluorescent enhancement by laser confocal fluorescence imaging, which corresponded to the increased ROS level in the Hela cells. When the Mn_3_O_4_ NPs were added, the fluorescence decreased obviously. And the more NPs were added, the more fluorescence decreased, which indicated the dose dependent intracellular ROS scavenging activities of Mn_3_O_4_ NPs (Fig. 5B and C).

### *In vivo* anti-inflammation

**Fig. 6:**
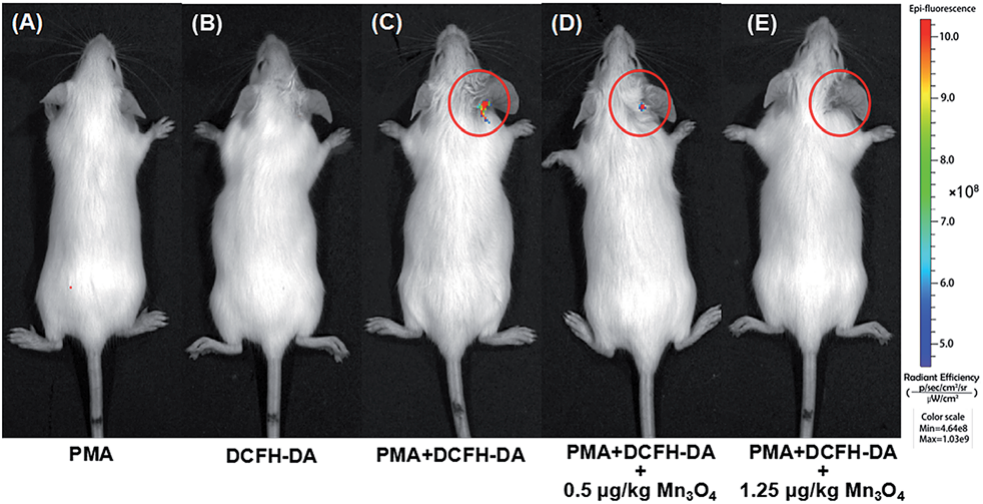
*In vivo* fluorescence imaging of mice with PMA-induced ear inflammation after treatment with (A) PMA, (B) DCFH-DA, (C) PMA and DCFH-DA, (D) PMA and DCFH-DA with 0.5 μg kg^–1^ Mn_3_O_4_ NPs, and (E) PMA and DCFH-DA with 1.25 μg kg^–1^ Mn_3_O_4_ NPs.

Encouraged by the above cellular results, an ear inflammation model was established to evaluate the anti-inflammation activity of Mn_3_O_4_ NPs *in vivo*. As displayed in Fig. S13,† the right ear of an experimental mouse appeared red and swollen after the topical application of phorbol 12-myristate 13-acetate (PMA) for 6 h, indicating a typical characteristic of inflammation. In order to study the *in vivo* ROS scavenging capability of Mn_3_O_4_ NPs, the inflamed ears were thereafter subcutaneously treated with Mn_3_O_4_ NPs at the dose of 0.5 μg kg^–1^ and 1.25 μg kg^–1^ for each mouse, respectively. As demonstrated in Fig. 6A and B, no obvious fluorescence was observed for mice after treatment with DCFH-DA or PMA only. In contrast, a strong fluorescence was observed in the PMA/DCFH-DA-treated ear (Fig. 6C), which indicated the elevated level of ROS due to the PMA induced inflammation. After treatment with Mn_3_O_4_ NPs, the fluorescence in the inflamed ear decreased obviously (Fig. 6D, E, and S14†). Hematoxylin and eosin (H&E) stained images of PMA induced inflammation ear were also obtained. As shown in Fig. S15B,† lymphocytes infiltration was observed obviously compared with the healthy mouse ear (Fig. S15A†). After treatment with Mn_3_O_4_ NPs, the symptom was alleviated (Fig. S15C and D†). Besides, as shown in Fig. S16,† H&E stained histology section of liver, spleen, and kidney demonstrated negligible toxicity of Mn_3_O_4_ NPs toward live tissues. These results indicated that the Mn_3_O_4_ NPs possessed efficient ROS scavenging capacity toward ear inflammation in live mice with safety.

## Conclusions

In summary, we synthesized Mn_3_O_4_ nanozymes with excellent ROS scavenging activity. Due to the SOD mimicking activity, they eliminated as much as nearly 75% ˙O_2_^–^, which was superior to CeO_2_ NPs. Besides, the Mn_3_O_4_ NPs could also catalyze the elimination of another two important ROS of H_2_O_2_ and ˙OH. *In vitro* experiments showed that the Mn_3_O_4_ NPs significantly scavenged intracellular ROS. Due to their excellent antioxidant activities *in vitro*, an inflammation model was established to evaluate the ROS scavenging ability of Mn_3_O_4_ NPs *in vivo*. The Mn_3_O_4_ NPs efficiently relieved the PMA-induced ear inflammation in live mice. This work not only demonstrated that Mn_3_O_4_ nanozymes possessed ROS scavenging activities, but also provided a promising therapeutic strategy for inflammation by using redox active nanozymes.

## Experimental

### Chemicals and materials

Manganese acetate (Mn(OAc)_2_) was purchased from Shanghai Meixing Chemical Reagent Co., Ltd. Hydrogen peroxide (H_2_O_2_, 30%) was obtained from Sinopharm Chemical Reagent Co., Ltd. Xanthine, xanthine oxidase, SOD from bovine erythrocytes, and phorbol 12-myristate 13-acetate (PMA) were supplied by Sigma-Aldrich. 2′,7′-Dichlorofluorescein diacetate (DCFH-DA) and Rosup were purchased from Beyotime Chemical Reagent Co., Ltd. 5,5-Dimethyl-1-pyridine N-oxide (DMPO) was from Nanjing Tongquan Chemical Reagent Co., Ltd. Hydroethidine (HE), salicylic acid (SA), and terephthalic acid (TA) were from Aladdin Chemical Reagent Co., Ltd. All chemical reagents were used as received without any further purification. All aqueous solutions were prepared with deionized water (18.2 MΩ cm, Millipore).

### Instrumentation

Transmission electron microscopy (TEM) images were obtained on a JEM-2100 microscope (JEOL, Japan) at an acceleration voltage of 200 kV. Powder X-ray diffraction (XRD) data was collected on a Rigaku Ultima diffractometer by using Cu Kα radiation. The diffractometer was operated at 40 kV and 40 mA with a scan rate of 5° min^–1^. Dynamic lighting scattering (DLS) and zeta potential distribution were measured on a Nanosizer ZS90 (Malvern). X-ray photoelectron spectroscopy (XPS) was collected using a PHI 5000 VersaProbe (Ulvac-Phi, Japan). All the measurements were carried out with reference to C 1s binding energy (285 eV) as the internal standard. UV-visible absorption spectra were recorded using a TU-1900 spectrophotometer (Beijing Purkinje General Instrument Co. Ltd., China). Nitrogen adsorption–desorption isotherms were measured at 77 K using a Quantachrome Autosorb-IQ-2C-TCD-VP analyzer, which were used to calculate the surface areas of the nanoparticles with the Brunauer–Emmett–Teller (BET) method. Photoluminescence spectra were measured on a Hitachi F-4600 spectrometer (Hitachi Co. Ltd., Japan). Electron paramagnetic resonance (EPR) detection was carried out in an EMX-10/12 EPR spectrometer (Bruker, Germany). A confocal laser scan microscopy was used for cell imaging, which consists of an epi-fluorescent microscope (IX-83, Olympus, with halogen lamp as the light source), a spinning disk confocal system (Andor), and an electron multiplying charge-coupled device (EMCCD) camera (Evolve 512, Photometrics). The *in vivo* fluorescence imaging of live mice was recorded on a PerkinElmer *In vivo* Imaging System with an excitation wavelength of 465 nm and an emission wavelength of 520 nm.

### Synthesis of Mn_3_O_4_ NPs

The Mn_3_O_4_ NPs were synthesized as follows.^46^ In a typical procedure, 1.225 g Mn(OAc)_2_·4H_2_O was dissolved in 60 mL anhydrous ethanol and magnetically stirred until fully dissolved. Then the mixture was transferred into a 100 mL Teflon-lined stainless steel autoclave for thermal treatment for 24 h at 120 °C. The products were washed by deionized water for three times after cooling down to room temperature, and the dark-brown precipitate was obtained.

### Synthesis of CeO_2_ NPs

The CeO_2_ NPs were synthesized according to our previous work.^48^

### SOD-like activity of Mn_3_O_4_ NPs

The SOD-like catalytic activity of Mn_3_O_4_ NPs was evaluated *via* two methods. Both of them monitored the amount of ˙O_2_^–^ scavenged. The more ˙O_2_^–^ scavenged, the higher SOD-like activity of Mn_3_O_4_ NPs was.

#### Method one

The amount of ˙O_2_^–^ scavenged was indirectly detected by measuring the fluorescent intensity of ethidium, which was the oxidation product of HE by ˙O_2_^–^. First, ˙O_2_^–^ was generated by mixing xanthine (0.6 mM) and xanthine oxidase (0.05 U mL^–1^) in phosphate buffer (0.1 M, pH 7.4) at 37 °C.^49^ After 40 min, the Mn_3_O_4_ NPs were added to the solution for another 40 min reaction, followed by adding HE (0.5 mg mL^–1^). Then the solution was vortexed and left undisturbed for 40 min before fluorescence measurement. Ethidium was excited at 470 nm and emitted at 610 nm. The scavenging percentage of ˙O_2_^–^ was calculated according to the following equation: elimination (%) = [(*F*_0_ – *F*)/*F*_0_] × 100%, where *F*_0_ and *F* were the fluorescent intensities of ethidium in the absence and presence of Mn_3_O_4_ NPs, respectively.

CeO_2_ NPs and natural SOD were also tested under the same condition for comparison.

#### Method two

EPR was used to directly detect ˙O_2_^–^ scavenging. The Mn_3_O_4_ NPs were added to the mixture of xanthine and xanthine oxidase to scavenge the generated ˙O_2_^–^. DMPO (100 mM) was employed to trap the unscavenged ˙O_2_^–^ by forming DMPO/˙OOH. DMSO was also added to enhance the stability of spin adduct.^50^

### Catalase-like activity of Mn_3_O_4_ NPs

The catalase-like activity of Mn_3_O_4_ NPs was evaluated by monitoring the catalytic elimination of H_2_O_2_ with Mn_3_O_4_ NPs using fluorescent and UV-visible absorption spectra.

#### Fluorescent method

H_2_O_2_ can decompose into ˙OH, which would react with TA to produce fluorescent 2-hydroxyterephthalic acid with an excitation wavelength of 320 nm and an emission peak at 425 nm.^51^ In the presence of catalase (or catalase mimics), H_2_O_2_ would decompose into H_2_O and O_2_ and could not generate fluorescent 2-hydroxyterephthalic acid. Therefore, by monitoring the fluorescent signal of 2-hydroxyterephthalic acid, the elimination of H_2_O_2_ could be investigated. After phosphate buffer (25 mM, pH 7.4) containing H_2_O_2_ (10 mM) and different concentrations of Mn_3_O_4_ NPs was vortexed vigorously and incubated at room temperature for 6 h, TA in DMF (0.5 mM) was added. Then the fluorescence of the mixture was measured.

#### Absorption method

The elimination of H_2_O_2_ was also detected by monitoring its characteristic absorbance at 240 nm using UV-visible absorption spectroscopy.

### Hydroxyl radical scavenging activity of Mn_3_O_4_ NPs

The ˙OH was generated through Fenton reaction of 1.8 mM FeSO_4_ and 5 mM H_2_O_2_ for 10 min. Two methods were applied to study the ˙OH scavenging activity of Mn_3_O_4_ NPs.

#### Method one

The amount of ˙OH scavenged was detected by measuring the characteristic absorbance of 2,3-dihydroxybenzoic acid at 510 nm, which was produced by reacting SA (1.8 mM) with ˙OH.

#### Method two

The scavenging of ˙OH was also detected by using EPR. DMPO was used as a spin trap to form DMPO/˙OH spin adduct, which yielded four characteristic lines with relative intensities of 1 : 2 : 2 : 1.

### Cytotoxicity assay

Hela cells were refreshed in high glucose DMEM culture medium containing 10% fetal bovine serum (FBS) and 1% penicillin–streptomycin (10 000 U mL^–1^) under the atmosphere of 5% CO_2_ at 37 °C. For cell viability assay, Hela cells were seeded into 96-well plates with a density of 8 × 10^3^ cells per well. The medium was refreshed after overnight incubation, followed by adding Mn_3_O_4_ NPs with different concentrations (0–10 μg mL^–1^). After incubating for another 24 h, the cells were washed by PBS for three times and the cell viability was determined by 3-(4,5-dimethyl-2-thiazolyl)-2,5-diphenyl-2-*H*-tetrazolium bromide (MTT) assay.

### Intracellular ROS scavenging detection

The intracellular level of ROS was monitored by using non-fluorescent DCFH-DA, which could penetrate through the cell membrane into cytosol and be hydrolyzed by intracellular esterase into DCFH. Then DCFH reacted with intracellular free radicals to produce fluorescent product DCF with excitation and emission wavelength at 488 nm and 520 nm, respectively. The whole procedure was performed as below: Hela cells were first seeded into 24-well plates and incubated for 24 h, followed by adding Rosup (0.5 mg mL^–1^) to produce intracellular ROS. After 30 min, the medium was refreshed three times and then Mn_3_O_4_ NPs with different concentrations were added for 1 h incubation. Subsequently, DCFH-DA (0.01 mM) in serum-free medium without phenol red was added after the cells were washed by PBS (pH 7.4) in triplicate. Cell nucleus was stained with Hoechst for confocal laser scan microscopy imaging.

### *In vivo* anti-inflammation

All the animal studies were approved by the Committee for Experimental Animals Welfare and Ethics of Nanjing Drum Tower Hospital, the Affiliated Hospital of Nanjing University Medical School. Kunming mice (20 g) were chosen for establishing an inflammation model.^52^ 50 μL of PMA (100 μg mL^–1^) acetone solution was topically applied on the right ear of each mouse to induce local ear inflammation. After 6 h induction, the mice were anesthetized with chloral hydrate and subcutaneously injected with Mn_3_O_4_ NPs (at a dose of 0.5 and 1.25 μg kg^–1^, respectively). After 30 min incubation, 50 μL of DCFH-DA (1 mM) was injected in a similar way. After another 30 min incubation, the whole body fluorescent images were recorded by a PerkinElmer *In vivo* Imaging System with an excitation wavelength of 488 nm and an emission wavelength of 520 nm. Mice without treatment and treated with PMA or DCFH-DA only were used as control.

### *In vivo* toxicity toward live tissues

*In vivo* toxicity of Mn_3_O_4_ NPs toward live tissues was evaluated by pathological observation of the tissues (including liver, spleen, and kidney) from the test groups. These tissues were collected and rinsed with deionized water, and then fixed in 10% neutral buffered formalin. The tissues were processed routinely, dried and embedded into paraffin, sectioned at a thickness of 4 mm, stained with hematoxylin and eosin (H&E), examined and photographed by optical microscopy.

## Conflicts of interest

There are no conflicts to declare.

## Supplementary Material

Supplementary information
