# Supplementary material for: ROS scavenging Mn3O4 nanozymes for in vivo anti-inflammation
Source: Chem Sci. 2018 Feb 16;9(11):2927–33. doi: 10.1039/c7sc05476a (PMC5915792; doi:10.1039/c7sc05476a)
Supplement: Supplementary file 1 [file SC-009-C7SC05476A-s001.pdf]

Electronic Supplementary Material (ESI) for Chemical Science.
This journal is © The Royal Society of Chemistry 2018

Supporting Information

ROS scavenging Mn_3O_4 nanozymes for *in vivo* anti-inflammation

Jia Yao,[a] Yuan Cheng,[a] Min Zhou,[a] Sheng Zhao,[a] Shichao Lin,[a] Xiaoyu Wang,[a] Jiangjiexing Wu,[a] Sirong Li,[a] and Hui Wei [a,b]

[a]Department of Biomedical Engineering, College of Engineering and Applied Sciences, Nanjing National Laboratory of Microstructures, Nanjing University, Nanjing, Jiangsu 210093, China.

[b]State Key Laboratory of Analytical Chemistry for Life Science, School of Chemistry and Chemical Engineering, Collaborative Innovation Center of Chemistry for Life Sciences, Nanjing University, Nanjing, Jiangsu 210023, China.

Email: weihui@nju.edu.cn; Fax: +86-25-83594648; Tel: +86-25-83593272; Web: http://weilab.nju.edu.cn.

Table of Contents

Figure S1. TEM images of (A) Mn_3O_4 NPs and (B) CeO_2 NPs.

Figure S2. Dynamic lighting scattering results of newly-prepared and two-month stored Mn_3O_4 NPs.

Figure S3. Zeta potential distribution of newly-prepared and two-month stored Mn_3O_4 NPs.

Figure S4. X-ray photoelectron spectrum of Mn_3O_4 NPs.

Figure S5. $^{\bullet}O_2^-$ elimination efficiency of Mn_3O_4 NPs, CeO_2 NPs, and natural SOD pretreated at 37 °C and 80 °C.

Figure S6. Temperature dependent $^{\bullet}O_2^-$ scavenging activities of Mn_3O_4 NPs and natural SOD.

Figure S7. (A) Fluorescent spectra of HE after reaction with xanthine and xanthine oxidase in the presence of Mn_3O_4 NPs (20 μg/mL) stored for different times. (B) $^{\bullet}O_2^-$ elimination of Mn_3O_4 NPs after different storage times.

Figure S8. EPR spectra of samples containing DMPO, xanthine, and xanthine oxidase in the absence (a) and presence of 2.5 μg/mL (b) or 5 μg/mL (c) Mn_3O_4 NPs. (d) EPR spectrum of DMPO only.

Figure S9. Comparison of the mass-based and the surface area normalized (*i.e.*, specific) SOD mimicking activities of Mn_3O_4 NPs and CeO_2 NPs.

Figure S10. H_2O_2 elimination efficiency of Mn_3O_4 NPs, CeO_2 NPs, and natural SOD pretreated at 37 °C and 80 °C.

Figure S11. Time evolution of absorbance of H_2O_2 at 240 nm during the catalytic elimination of H_2O_2 by Mn_3O_4 NPs.

Figure S12. EPR spectra of DMPO, Fe^{2+}, and H_2O_2 in the absence (a) and presence of 2.5 μg/mL (b) or 5 μg/mL (c) Mn_3O_4 NPs. (d) EPR spectrum of DMPO only.

Figure S13. Photograph of a live mouse, showing an inflammatory ear (right) induced by PMA.

Figure S14. Total radiant efficiency of the fluorescence images acquired in live mice after different treatments.

Figure S15. H&E stained images of (A) healthy mouse ear, (B) PMA induced inflammation ear, (C) PMA induced inflammation ear treated with 0.5 μg/kg Mn_3O_4 NPs, and (D) PMA induced inflammation ear treated with 1.25 μg/kg Mn_3O_4 NPs.

Figure S16. H&E stained histology sections of different tissues (liver, spleen, and kidney). Mn1 and Mn2 represent low (0.5 μg/kg) and high concentration (1.25 μg/kg) of Mn_3O_4 NPs, respectively.

Table S1. BET surface area of CeO_2 and Mn_3O_4 nanozymes.

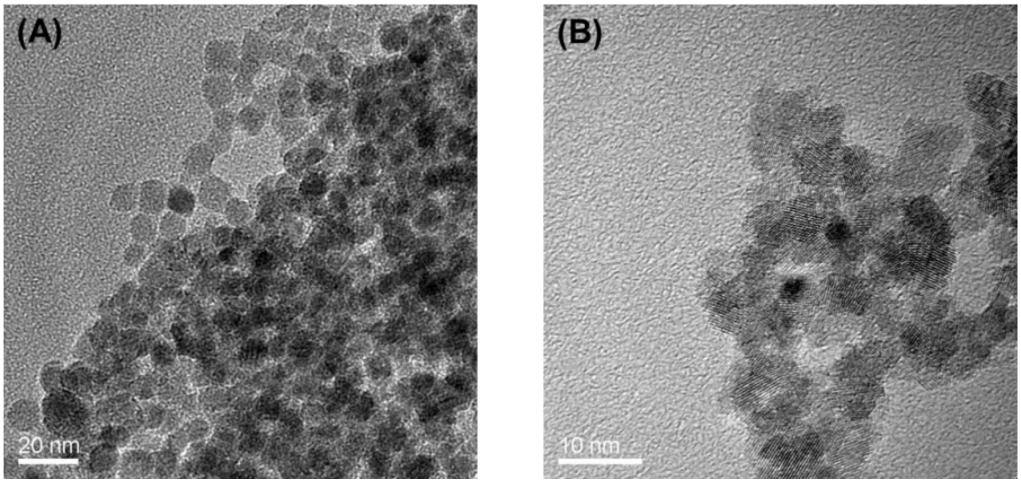

Figure S1. TEM images of (A) Mn_3O_4 NPs and (B) CeO_2 NPs.

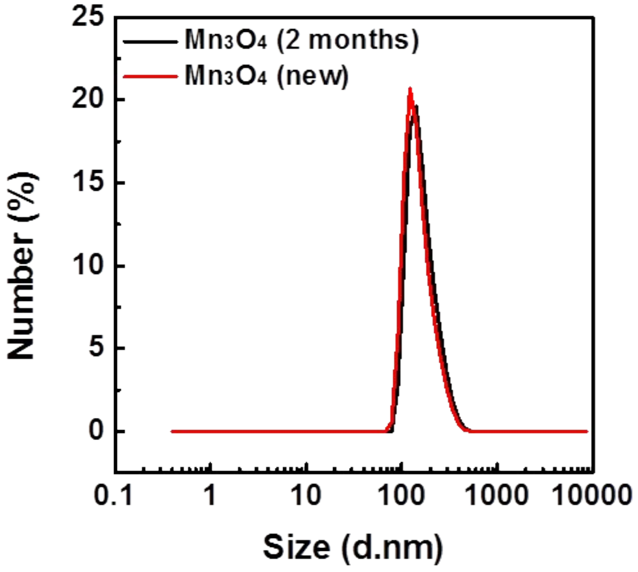

Figure S2. Dynamic lighting scattering results of newly-prepared and two-month stored Mn_3O_4 NPs.

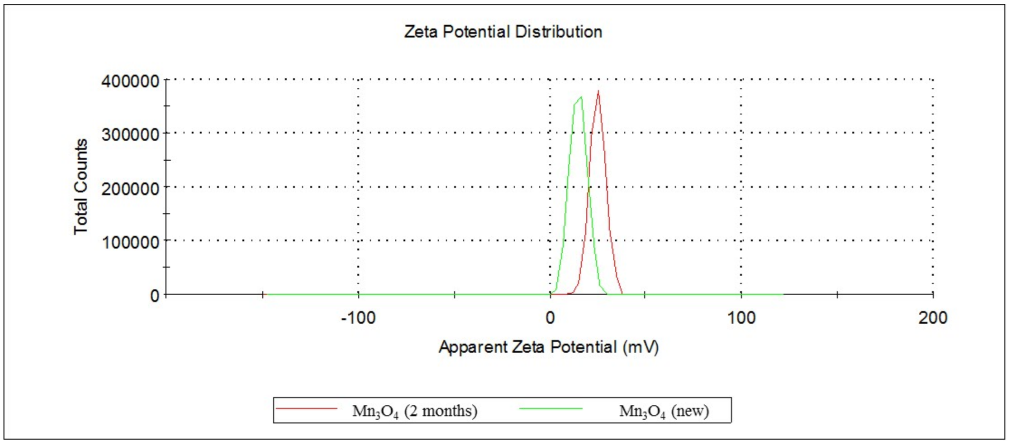

Figure S3. Zeta potential distribution of newly-prepared and two-month stored Mn_3O_4 NPs.

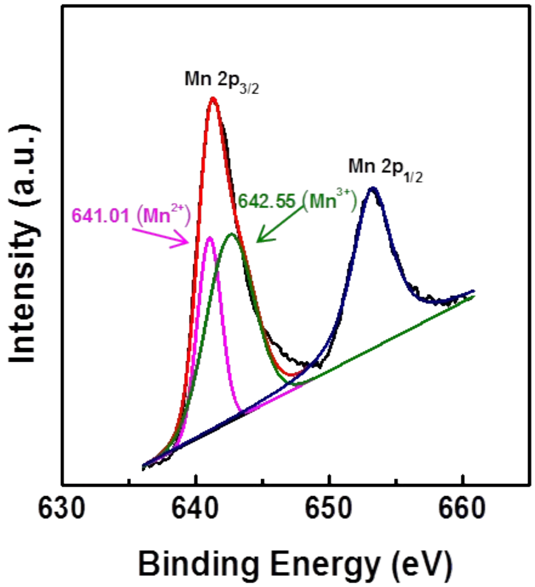

Figure S4. X-ray photoelectron spectrum of Mn_3O_4 NPs.

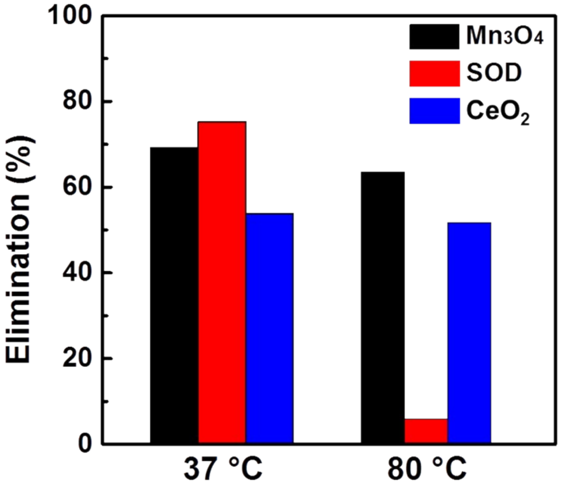

Figure S5. $^{\bullet}O_2^-$ elimination efficiency of Mn_3O_4 NPs, CeO_2 NPs, and natural SOD pretreated at 37 °C and 80 °C.

As shown in Figure S5, all of the three catalysts (*i.e.*, Mn_3O_4 NPs, CeO_2 NPs, and natural SOD) exhibited good $^{\bullet}O_2^-$ scavenging activity. However, after pretreatment in 80 °C water bath for 1 h, natural SOD almost lost its activity, while the Mn_3O_4 NPs and CeO_2 NPs still retained high $^{\bullet}O_2^-$ scavenging activity. This result demonstrated that the excellent thermal stability of Mn_3O_4 and CeO_2 nanozymes.

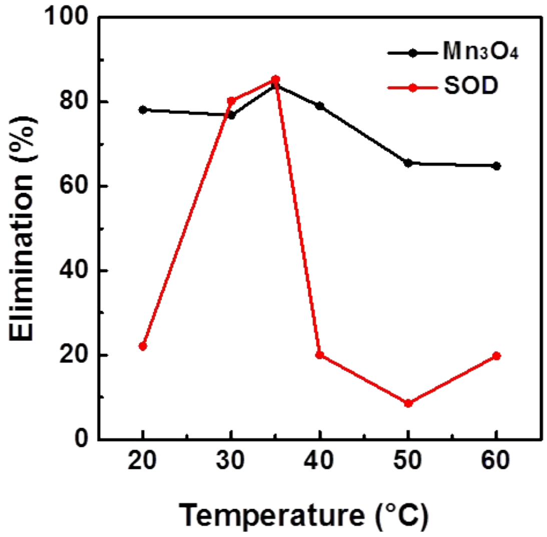

Figure S6. Temperature dependent $^{\bullet}O_2^-$ scavenging activities of Mn_3O_4 NPs and natural SOD.

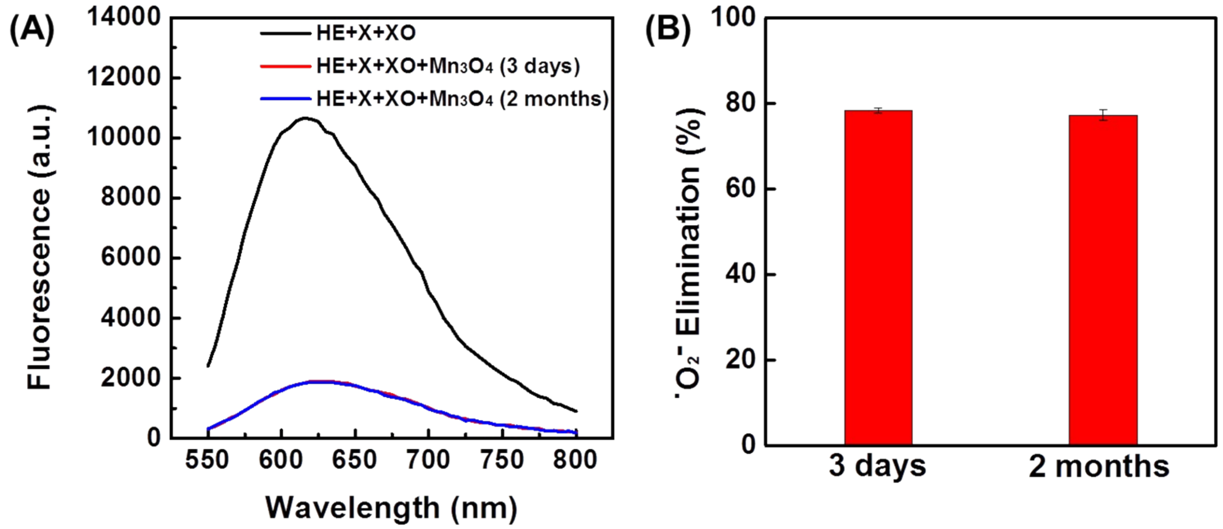

Figure S7. (A) Fluorescent spectra of HE after reaction with xanthine and xanthine oxidase in the presence of Mn_3O_4 NPs (20 μg/mL) stored for different times. (B) $^{\bullet}O_2^-$ elimination of Mn_3O_4 NPs after different storage times.

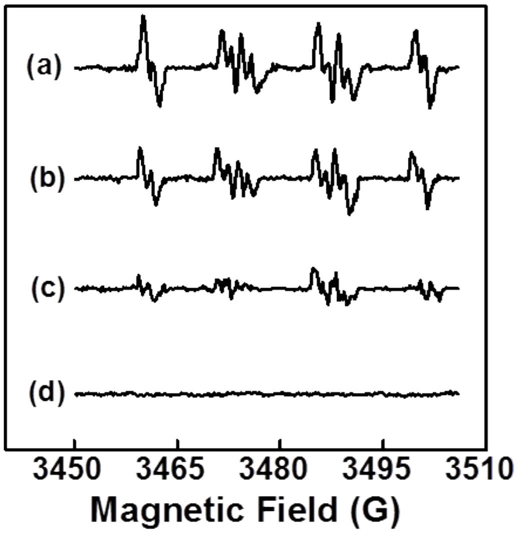

Figure S8. EPR spectra of samples containing DMPO, xanthine, and xanthine oxidase in the absence (a) and presence of 2.5 μg/mL (b) or 5 μg/mL (c) Mn_3O_4 NPs. (d) EPR spectrum of DMPO only.

$^{\bullet}O_2^-$ generated from xanthine and xanthine oxidase was trapped by DMOP, which was subsequently detected by EPR (Figure S8a). The characteristic EPR spectrum of DMPO/$^{\bullet}$OOH gradually decreased with the increase of Mn_3O_4 concentration, demonstrating the $^{\bullet}O_2^-$ scavenging activity of Mn_3O_4 NPs.

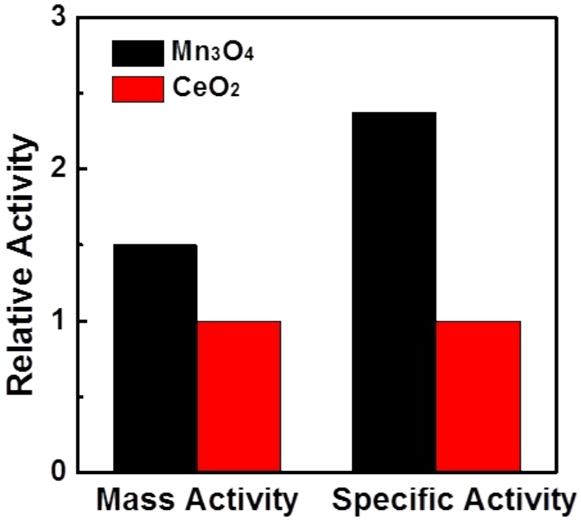

Figure S9. Comparison of the mass-based and the surface area normalized (*i.e.*, specific) SOD mimicking activities of Mn_3O_4 NPs and CeO_2 NPs.

To understand the effect of surface area on the SOD mimicking activity, we investigated the surface area normalized (*i.e.*, specific) SOD mimicking activity of Mn_3O_4 NPs and CeO_2 NPs. As shown in Table S1, the surface area of CeO_2 NPs was 166.43 m^2/g, which was higher than that of Mn_3O_4 NPs. Therefore, compared with CeO_2 NPs, Mn_3O_4 NPs showed even better specific SOD mimicking activity than the mass based activity (~2.4-fold enhancement vs ~1.5-fold enhancement) (Figure S9).

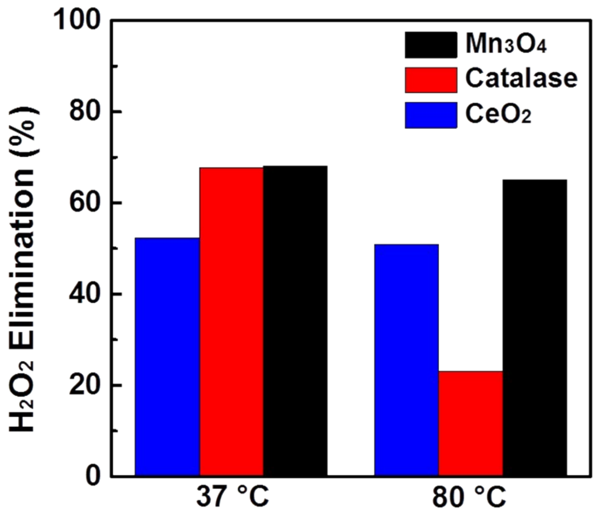

Figure S10. H_2O_2 elimination efficiency of Mn_3O_4 NPs, CeO_2 NPs, and natural SOD pretreated at 37 °C and 80 °C.

As shown in Figure S10, all of the three catalysts (*i.e.*, Mn_3O_4 NPs, CeO_2 NPs, and natural SOD) exhibited good H_2O_2 decomposition activity (*i.e.*, catalase mimicking activity). However, after pretreatment in 80 °C water bath for 1 h, natural catalase almost lost its activity, while the Mn_3O_4 NPs and CeO_2 NPs still retained high H_2O_2 decomposition activity. This result demonstrated the better thermal stability of Mn_3O_4 nanozymes than natural catalase.

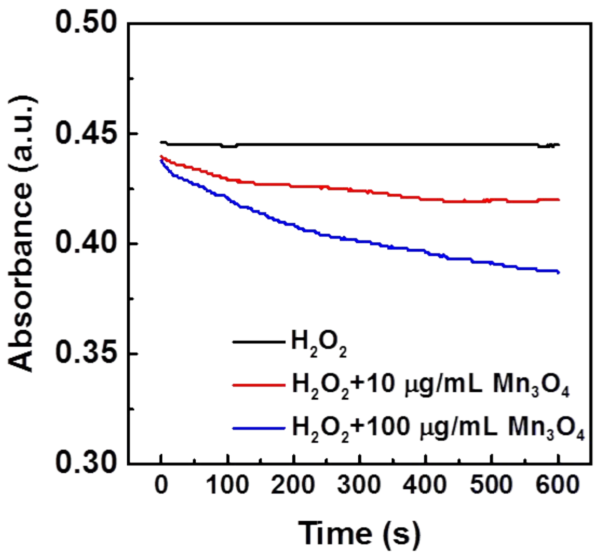

Figure S11. Time evolution of absorbance of H_2O_2 at 240 nm during the catalytic elimination of H_2O_2 by Mn_3O_4 NPs.

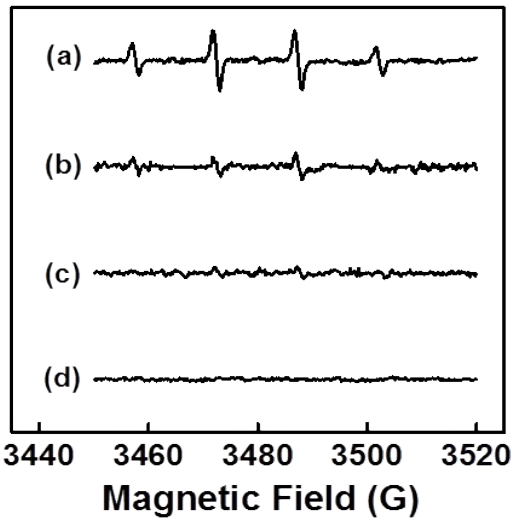

Figure S12. EPR spectra of DMPO, Fe^{2+}, and H_2O_2 in the absence (a) and presence of 2.5 µg/mL (b) or 5 µg/mL (c) Mn_3O_4 NPs. (d) EPR spectrum of DMPO only.

$^{\bullet}OH$ generated from Fe^{2+} and H_2O_2 was trapped by DMOP, which was subsequently detected by EPR (Figure S12a). The characteristic EPR spectrum of DMPO/$^{\bullet}OH$ gradually decreased with the increase of Mn_3O_4 concentration, demonstrating the $^{\bullet}OH$ scavenging activity of Mn_3O_4 NPs.

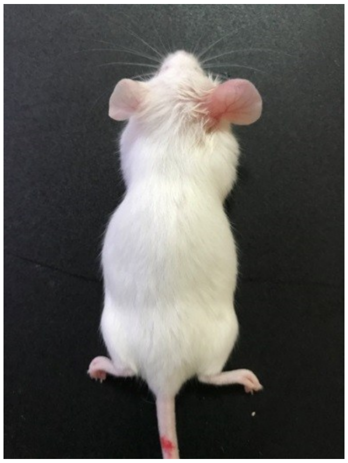

Figure S13. Photograph of a live mouse, showing an inflammatory ear (right) induced by PMA.

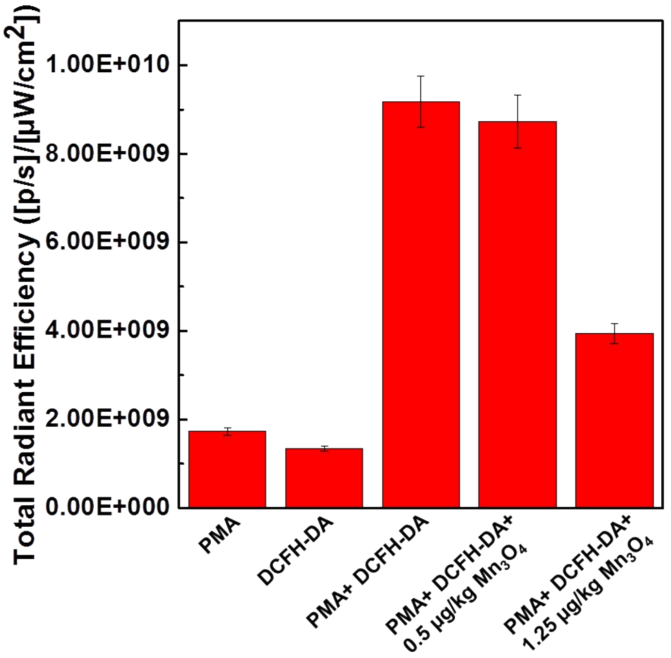

Figure S14. Total radiant efficiency of the fluorescence images acquired in live mice after different treatments.

The total radiant efficiency was calculated by living image 4.5.1 software system. The total radiant efficiency of mice treated with PMA or DCFH-DA only was much lower than those treated with both PMA and DCFH-DA. The radiant efficiency gradually decreased with the increase of Mn_3O_4 concentration, demonstrating the ROS scavenging activity of Mn_3O_4 NPs *in vivo*.

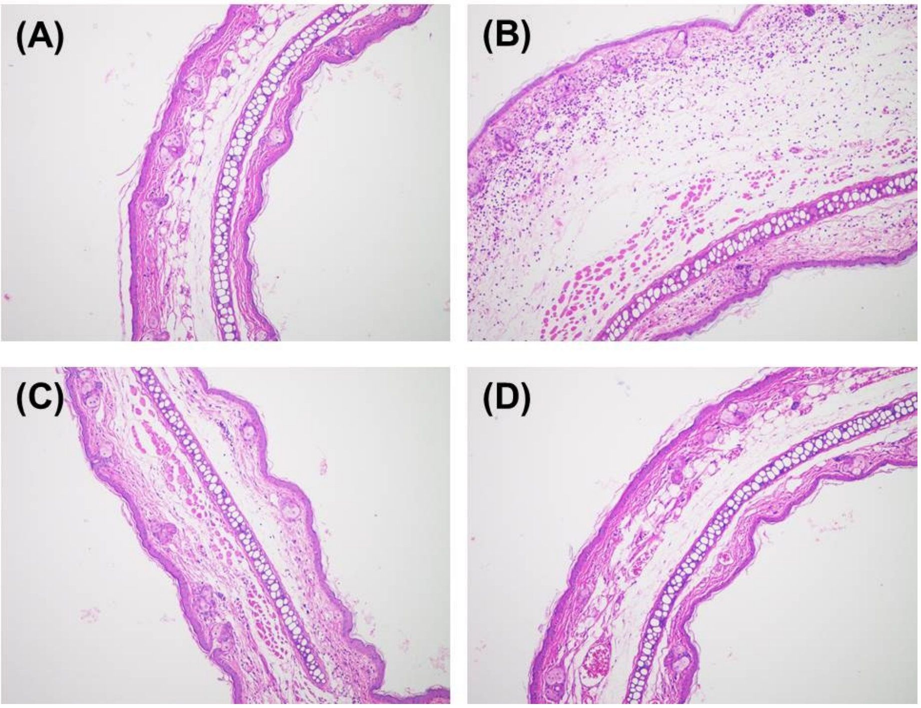

Figure S15. H&E stained images of (A) healthy mouse ear, (B) PMA induced inflammation ear, (C) PMA induced inflammation ear treated with 0.5 μg/kg Mn_3O_4 NPs, and (D) PMA induced inflammation ear treated with 1.25 μg/kg Mn_3O_4 NPs.

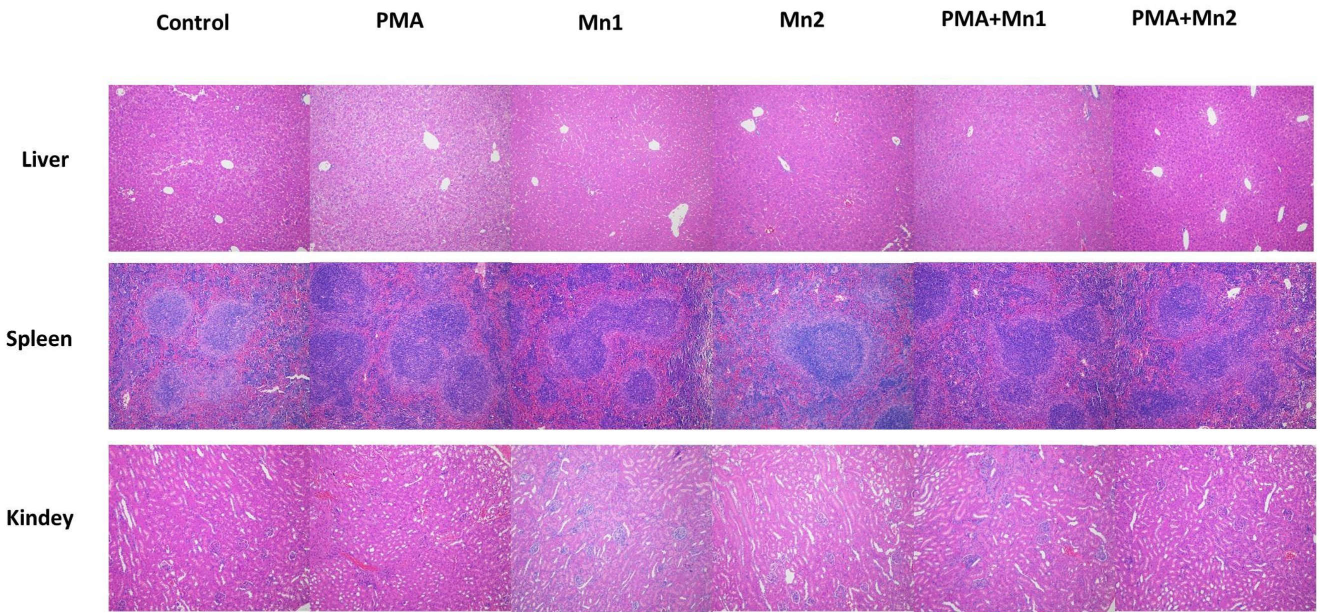

Figure S16. H&E stained histology sections of different tissues (liver, spleen, and kidney). Mn1 and Mn2 represent low (0.5 μg/kg) and high concentration (1.25 μg/kg) of Mn_3O_4 NPs, respectively.

Table S1. BET surface area of CeO_2 and Mn_3O_4 nanozymes.

**Nanozymes**	**BET surface area (m^2/g)**
Mn_3O_4 NPs	105.13
CeO_2 NPs	166.43
